# Analysis of risk factors compromising embryo quality and quantity in patients with endometriosis

**DOI:** 10.3389/fmed.2026.1808405

**Published:** 2026-04-23

**Authors:** Hanhong Lai, Guolu Yang, Jun Zhang

**Affiliations:** 1 Department of Reproductive Medicine, Shenzhen People's Hospital, The Second Clinical Medical College, Jinan University, Shenzhen, China; 2 Department of Reproductive Medicine, The Second Clinical Medical College of Jinan University, Shenzhen People's Hospital, Shenzhen, Guangdong, China

**Keywords:** assisted reproductive technology, embryo quality, endometriosis, IVF/ICSI, nomogram

## Abstract

**Purpose:**

This study aims to analyze the clinical factors affecting embryo quality and quantity in endometriosis patients undergoing *in vitro* fertilization/intracytoplasmic sperm injection and embryo transfer (IVF/ICSI-ET), thereby providing a reference for the clinical prediction of embryonic development outcomes in this patient population.

**Methods:**

Univariate logistic regression was performed to select variables (*P* < 0.05), which were then entered into a multivariate logistic regression model to develop a predictive model for the likelihood of obtaining a sufficient number of high-quality embryos. A predictive nomogram was constructed based on the findings from the univariate and multivariate analyses. Restricted cubic splines were employed to identify potential non-linear relationships and threshold effects concerning ovarian reserve-related parameters.

**Results:**

The model demonstrated robust discriminative ability, with area under the curve values of 0.765 (95% CI: 0.749–0.769) in the training set and 0.776 (95% CI: 0.747–0.789) in the validation set. Subgroup analyses further revealed that endometriomas represent a pivotal phenotype adversely affecting both the yield of oocytes and the rate of high-quality embryos.

**Conclusion:**

Anti-Müllerian Hormone level, Antral Follicle Count, the presence of bilateral endometriomas, and the utilization of gonadotropin-releasing hormone (GnRH) agonist pre-treatment serve as robust indicators for predicting oocyte yield in patients with endometriosis. The nomogram incorporating these factors provides a practical tool for the early prognostication of ovarian response, thereby offering valuable insights for formulating personalized treatment strategies. GnRH agonist administration demonstrates efficacy in improving clinical outcomes of IVF/ICSI cycles in these patients.

## 1 Introduction

Endometriosis is an estrogen-dependent chronic inflammatory condition characterized by the ectopic growth of endometrial glands and stroma outside the uterine cavity (1, 2). It represents a major etiology of infertility among women of reproductive age. It is reported that approximately 30% of endometriosis patients experience infertility, while 40–50% of infertility cases are diagnosed with endometriosis (3). The disease manifests with heterogeneous phenotypes, primarily classified into peritoneal endometriosis, ovarian endometrioma (OMA), and deep infiltrating endometriosis (DIE) (4). *In vitro* fertilization/intracytoplasmic sperm injection and embryo transfer (IVF/ICSI-ET) offers a viable treatment option for endometriosis-associated infertility by bypassing the inflammatory environment. Although the precise pathogenic mechanisms remain incompletely elucidated, impaired oocyte quality and compromised embryonic developmental potential are considered key contributors to reduced fertility. Several studies indicate that endometriosis, particularly OMA, adversely affects ovarian reserve and leads to a diminished ovarian response to stimulation (5–7). Patients with advanced-stage endometriosis yield fewer oocytes and exhibit lower cumulative pregnancy rates compared to those without the condition (8). Those with OMA also require higher gonadotropin doses and produce fewer oocytes, MII oocytes, and viable embryos. Some studies suggest that pregnancy rates in endometriosis patients are comparable to those in normoresponders, implying no detrimental effect of endometriosis on IVF/ICSI outcomes (9, 10). However, pregnancy rates per transfer are more closely related to endometrial receptivity than to ovarian response. The work of Simon et al. (11) demonstrated that endometriosis patients receiving donor oocytes achieved pregnancy rates similar to controls, supporting the hypothesis that compromised embryo quality may be a critical factor in endometriosis-related infertility. Other studies have also observed that, aside from reduced oocyte yield, live birth rates in endometriosis patients are comparable to those with tubal factor infertility (10, 12, 13). Therefore, enhancing both the quantity and quality of embryos remains a central challenge. A greater number of high-quality embryos increases the number of available transfer cycles, thereby improving cumulative pregnancy rates.

Furthermore, several studies report that ovarian reserve and oocyte retrieval numbers decline in patients with endometriomas following cystectomy (14, 15). Given the potential adverse impact of surgery on fertility, the decision to perform surgical intervention in endometriosis patients seeking ART remains clinically contentious. Current European Society of Human Reproduction and Embryology guidelines do not recommend routine surgery for asymptomatic endometriosis patients undergoing infertility treatment, nor do they advocate histological confirmation for diagnosis (16). Another area of ongoing debate concerns the use of GnRH agonists. A 2006 systematic review by Salam et al. indicated that pretreatment with GnRH agonists for 3–6 months could improve clinical pregnancy and live birth rates in endometriosis patients (17). Similar conclusions were drawn in a meta-analysis by Cao et al. (18). In contrast, a larger meta-analysis by Georgiou et al. (19) suggested that ultra-long protocols did not increase oocyte or embryo yield and were associated with reduced live birth rates. Thus, no consensus exists on the optimal stimulation protocol for endometriosis. The risk of poor ovarian response with GnRH agonists also implies that gonadotropin-releasing hormone antagonists (GnRH-A) may be a reasonable alternative for patients with compromised ovarian reserve due to OMA or prior cystectomy.

While recent advances have refined our understanding of endometriosis-associated infertility, individualized treatment strategies hold promise for further improving IVF/ICSI outcomes. Nevertheless, the specific impact of different endometriosis phenotypes on oocyte and embryo quantity and quality, as well as the precise risk factors contributing to impaired embryogenesis, remain incompletely defined. This study therefore aims to develop a predictive model for the likelihood of obtaining a sufficient number of high-quality embryos and explore clinical management strategies for endometriosis, thereby facilitating the formulation of personalized treatment plans.

## 2 Methods

### 2.1 Study population and ethical considerations

This study enrolled 562 patients with endometriosis who underwent IVF/ICSI-ET treatment at Shenzhen People's Hospital between January 2016 and June 2024. The study protocol was reviewed and approved by the Hospital's Ethics Committee (Approval No: LL-KY-2025002-02). Inclusion criteria were as follows: (1) Prior laparoscopic and/or hysteroscopic diagnosis of pelvic endometriosis confirmed by histopathological examination. (2) Clinical confirmation of endometriosis via physical examination, transvaginal ultrasonography, and/or magnetic resonance imaging. (3) Undergoing the first cycle of IVF treatment. Exclusion criteria comprised: (1) Coexisting ovarian tumors of a process of ovarian surgery. (2) Recurrence of ovarian endometrioma following previous cystectomy. (3) Diagnosis of polycystic ovary syndrome. (4) Chromosomal abnormalities in either partner. (5) History of recurrent miscarriage. (6) Metabolic syndromes such as diabetes mellitus of insulin resistance. (7) Concurrent autoimmune or rheumatic diseases.

### 2.2 Grouping method

The study cohort was randomly allocated into a training set (*N* = 401) and a validation set (*N* = 161) at a 7:3 ratio using a computer-generated random number sequence. The training set was utilized for variable selection and predictive model development, while the validation set served for external validation of the constructed model. To investigate phenotype-specific effects, patients were stratified into three subgroups: an OMA group, a DIE group, and a combined OMA with DIE group. Furthermore, within each phenotypic subgroup, patients were categorized based on their controlled ovarian stimulation (COS) protocol into corresponding GnRH agonist down-regulation and non-down-regulation groups. Additionally, patients presenting with ovarian endometrioma were subdivided into a surgical group and a non-surgical group according to whether they had undergone cystectomy prior to IVF/ICSI treatment.

### 2.3 Controlled ovarian stimulation protocol

During IVF treatment, ovarian stimulation protocols are primarily categorized into down-regulation and non-down-regulation regimens. Down-regulation protocols include the ultra-long protocol, the follicular phase long protocol, and the long-acting long protocol. Non-down-regulation regimens consist of the antagonist protocol, modified antagonist protocol, and mild stimulation protocol. The selection of a specific protocol is individualized based on the patient's ovarian reserve, age, and the severity of endometriosis The criteria for successful pituitary down-regulation were defined as the presence of follicles with a diameter of < 5 mm, an endometrial thickness of < 5 mm, and serum levels of estradiol (E2) < 50 pg/ml and luteinizing hormone (LH) < 5 IU/L. Recombinant human follicle-stimulating hormone (r-hFSH; Gonal-f, 75 IU/vial, Merck Europe B.V. / Livzon Group, 75 IU/vial) was administered at an initial dose of 150 or 225 IU, with subsequent adjustments made according to follicular growth. Ovarian response was monitored through serial serum hormone measurements and transvaginal ultrasonography. When the leading follicle reached a diameter of 12 mm, and/or serum estradiol levels exceeded 150 pg/ml and/or LH > 10 IU/L, daily administration of a GnRH antagonist (0.25 mg) was initiated to prevent a premature LH surge. Final oocyte maturation was triggered with recombinant human chorionic gonadotropin (hCG, 10,000 IU, Livzon Group) or a dual trigger consisting of hCG (8,000 IU) plus 0.2 mg of a GnRH agonist (Decapeptyl, 0.1 mg/vial, Ferring Pharmaceuticals) when at least three follicles reached > 18 mm or one follicle exceeded > 19 mm in diameter, with the specific trigger strategy determined by the patient's risk of developing ovarian hyperstimulation syndrome (OHSS). Oocyte retrieval was performed 36–37 h post-trigger, aspirating all follicles larger than 10 mm in diameter. Fertilization was accomplished via conventional insemination or intracytoplasmic sperm injection (ICSI), as clinically indicated.

### 2.4 Embryo culture and assessment

The embryo culture strategy adopted in this study was designed to secure transferable embryos while subjecting the remainder to extended culture for blastocyst development. Fertilization was confirmed on Day 1 (16–20 h post-insemination) by examining oocytes for the presence of two pronuclei. Subsequent embryonic development was evaluated at specific timepoints: Day 2 (44±1 h), Day 3 (68±1 h), and Day 5/6 (116±2 / 140±2 h). Assessments focused on cleavage stage-specific parameters, including blastomere number, size, and fragmentation. Cleavage-stage embryos were graded using Peter's scoring system. Blastocysts were assessed on Day 5 or 6 according to the Gardner blastocyst grading system, which evaluates the degree of expansion, the quality of the inner cell mass (ICM), and the morphology of the trophectoderm (TE). For the purpose of this study, a high-quality embryo was defined as a cleavage-stage embryo with a Peter's grade I or II, or a blastocyst scoring ≥ 4BB according to the Gardner criteria (20).

### 2.5 Study variables and outcome measures

**Figure 1:**
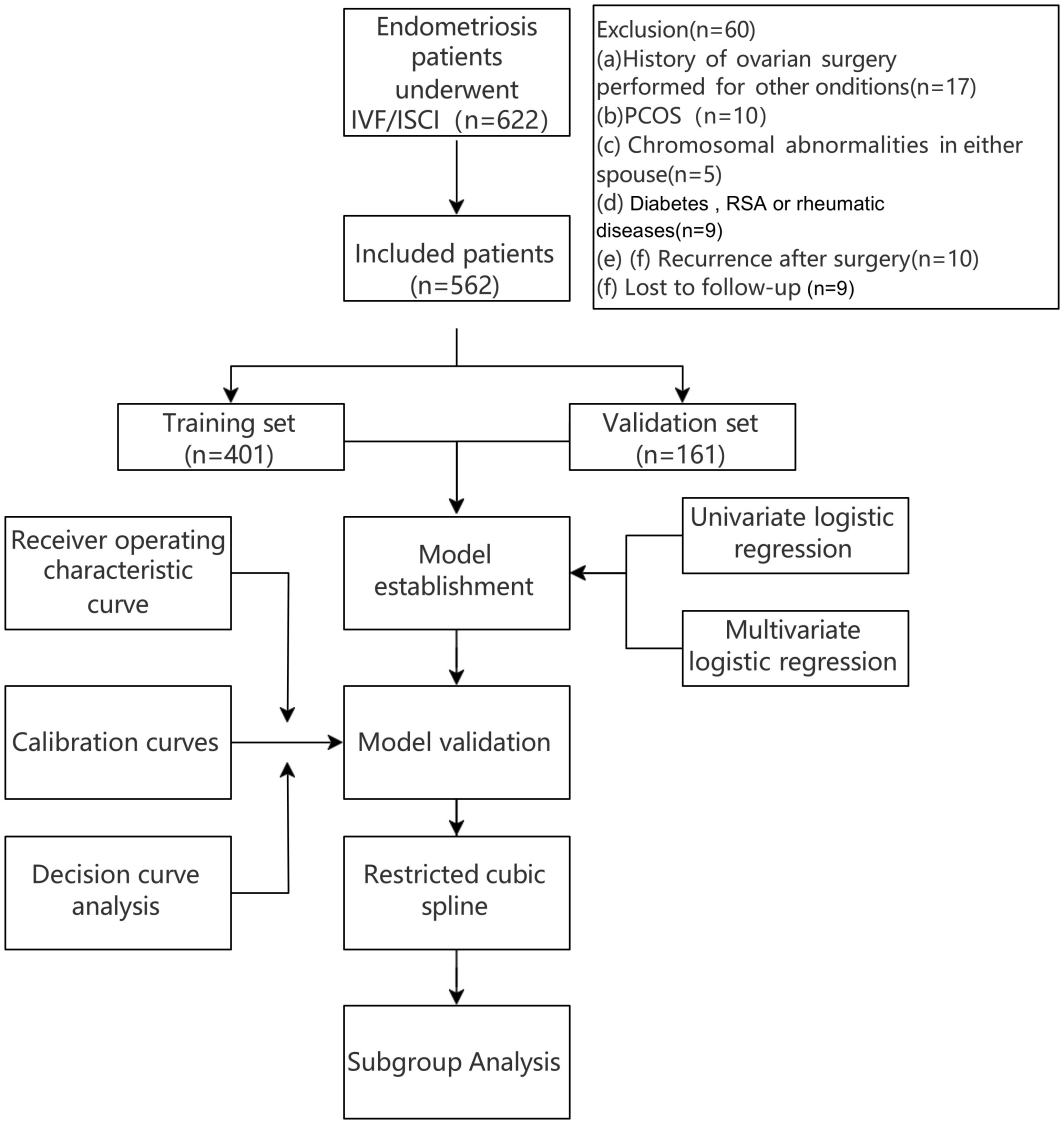
The flow chart of the study.

The collected baseline clinical characteristics included patient age, body mass index (BMI), duration of infertility, Antral Follicle Count (AFC), serum Anti-Müllerian Hormone level (AMH) level, and baseline sex hormone profiles. Additionally, the following clinical proportions were documented: the distribution of controlled ovarian stimulation protocols, types of infertility, the proportion of patients with a history of endometriosis-related surgery, the incidence of bilateral ovarian endometriomas, and the prevalence of coexisting male factors. The evaluated laboratory and embryological parameters encompassed: the number of oocytes retrieved, fertilization rate, two pronuclei (2PN) rate, cleavage rate, number of MII oocytes, Day 3 usable embryo rate, Day 3 high-quality embryo rate, usable blastocyst rate, and high-quality blastocyst rate. The primary outcome variable for the construction of the nomogram was defined as the ability to obtain at least three high-quality embryos in the first ovarian stimulation cycle (Figure 1).

### 2.6 Statistical analysis

All statistical analyses were performed using SPSS 20.0 (IBM Corp.) and R software. The normality of continuous variables was assessed using the Kolmogorov-Smirnov test, and homogeneity of variances was evaluated with Levene's test. The sample size for the nomogram was calculated using the “pmsampsize” package in R, and interaction effects were analyzed with the “rms” package. The outcome variable was defined as the attainment of three high-quality embryos. Univariate binary logistic regression was initially employed to identify clinical factors significantly associated with the outcome variable, with effects estimated using odds ratios (OR) and their 95% confidence intervals (CI). Multicollinearity among independent variables was examined prior to multivariate modeling. Variables yielding a *P*-value < 0.05 in the univariate analysis were subsequently incorporated into a multivariate logistic regression model using a stepwise selection procedure to identify independent risk factors influencing embryo quality in endometriosis patients. A predictive nomogram was then constructed based on the significant predictors identified in these analyses. The discriminative ability of the nomogram was quantified by the area under the receiver operating characteristic curve. Calibration accuracy was evaluated using calibration plots, and clinical utility was assessed via decision curve analysis. Interaction terms were introduced into the model, and their significance was tested using *t*-tests and *F*-tests. The bootstrap method was employed to estimate the 95% CI for the AUC. Restricted cubic splines (RCS) were applied to explore potential non-linear relationships and threshold effects between continuous predictors and the outcome. Normally distributed continuous data are presented as mean ± standard deviation (x ± s), while non-normally distributed data are summarized as median (Q1, Q3). Categorical variables are expressed as frequencies (%). Propensity score matching was utilized to balance baseline characteristics where applicable. Group comparisons were conducted using the Mann-Whitney U test or independent samples *t*-test for continuous variables, as appropriate. The Chi-square test and/or Fisher's exact test were used for categorical variables. The Cochran-Mantel-Haenszel test was applied to assess the confounding effect of stratified factors in categorical data analysis. Comparisons among three or more groups for continuous variables were performed using the Kruskal-Wallis test. A two-sided *P*-value < 0.05 was considered statistically significant for all tests unless otherwise specified.

## 3 Results

### 3.1 Univariate and multivariate analysis

**Figure 2:**
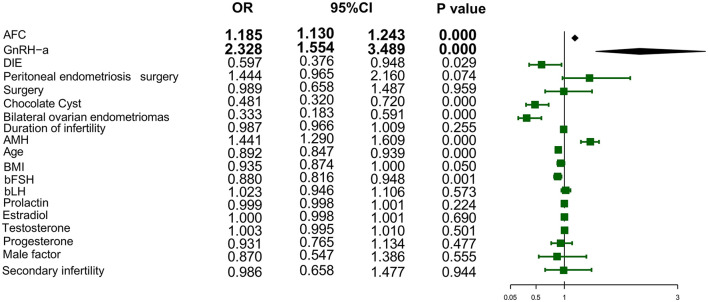
Forest plot of univariate analysis outcomes associated with embryo yield in patients with endometriosis. BMI, Body Mass Index; AFC, antral follicle counts; GnRH, Gonadotropin-releasing hormone; AMH, anti-Müllerian hormone; bFSH, basal Follicle Stimulating Hormone; bLH, basal Luteinizing hormone; DIE, deep infiltrating endometriosis.

A total of 562 eligible patients were randomly allocated into a training set (*n* = 401) and a validation set (*n* = 161) at a 7:3 ratio. Univariate analysis identified the following factors as significantly associated (*P* < 0.05) with embryo quality in patients with endometriosis: AFC, anti-Müllerian hormone AMH level, the presence of ovarian endometrioma, the presence of bilateral ovarian endometriomas, the presence of DIE, age, the coexistence of male factors, the controlled ovarian stimulation protocol, and the type of infertility (Figure 2). These significant factors were subsequently entered into a multivariate logistic regression model using a stepwise selection method. The analysis identified AFC, AMH level, the presence of bilateral ovarian endometriomas, and the ovarian stimulation protocol as the optimal set of independent predictors for the final model (Table 1). Multicollinearity assessment for all variables included in the multivariate analysis confirmed the absence of significant collinearity, with no covariates exhibiting a tolerance < 0.5 or a variance inflation factor (VIF) > 5.

**Table 1:** Multivariate analysis of influencing factors.

Characteristics	B	SE	OR	95% CI	*p* value
AFC	0.112	0.028	1.119	1.060–1.181	< 0.001
AMH	0.207	0.062	1.230	1.088–1.390	0.001
GnRH–agonist	0.465	0.233	1.592	1.008–2.516	0.047
Bilateral ovarian endometriomas	−0.700	0.340	0.497	0.255–0.967	0.026

AMH, anti-Müllerian hormone; AFC, antral follicle counts; GnRH, Gonadotropin-releasing hormone.

### 3.2 Nomogram construction and validation

**Figure 3:**
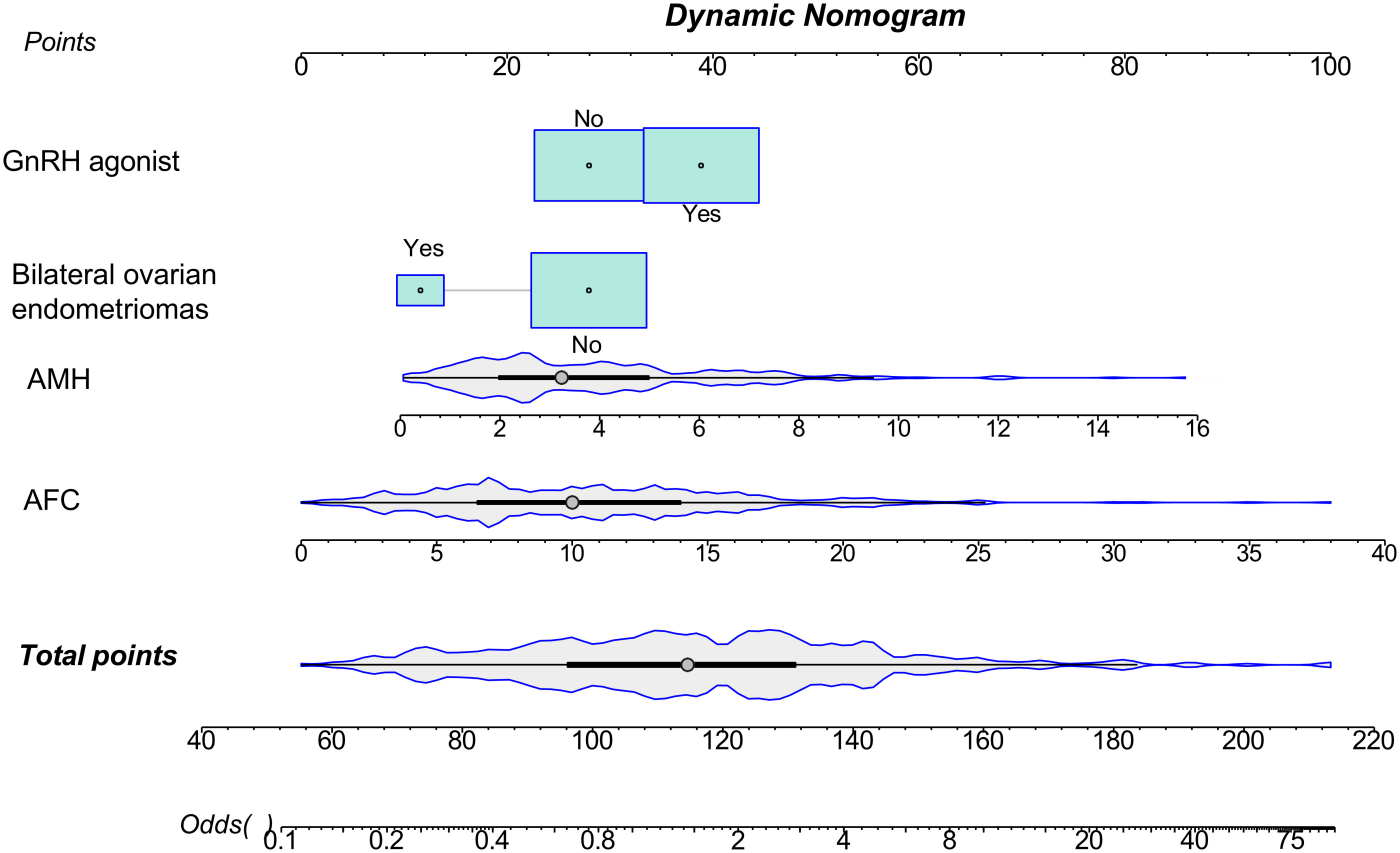
Nomogram to predict embryo yield after IVF in patients with endometriosis. To use the nomogram, locate the patient's AFC value on the corresponding axis, and draw a vertical line upward to the “Points” axis at the top to obtain the assigned score for that variable. Repeat this process for each predictor—AMH value, presence of bilateral ovarian endometriomas (Yes or No), and type of GnRH agonist protocol used—by drawing a line from each variable axis to the “Points” axis. Sum the scores for all predictors to obtain a total point value. Then, locate the total point value on the “Total points” axis, and draw a line downward to the corresponding value on the “Odds” axis to estimate the likelihood.

**Figure 4:**
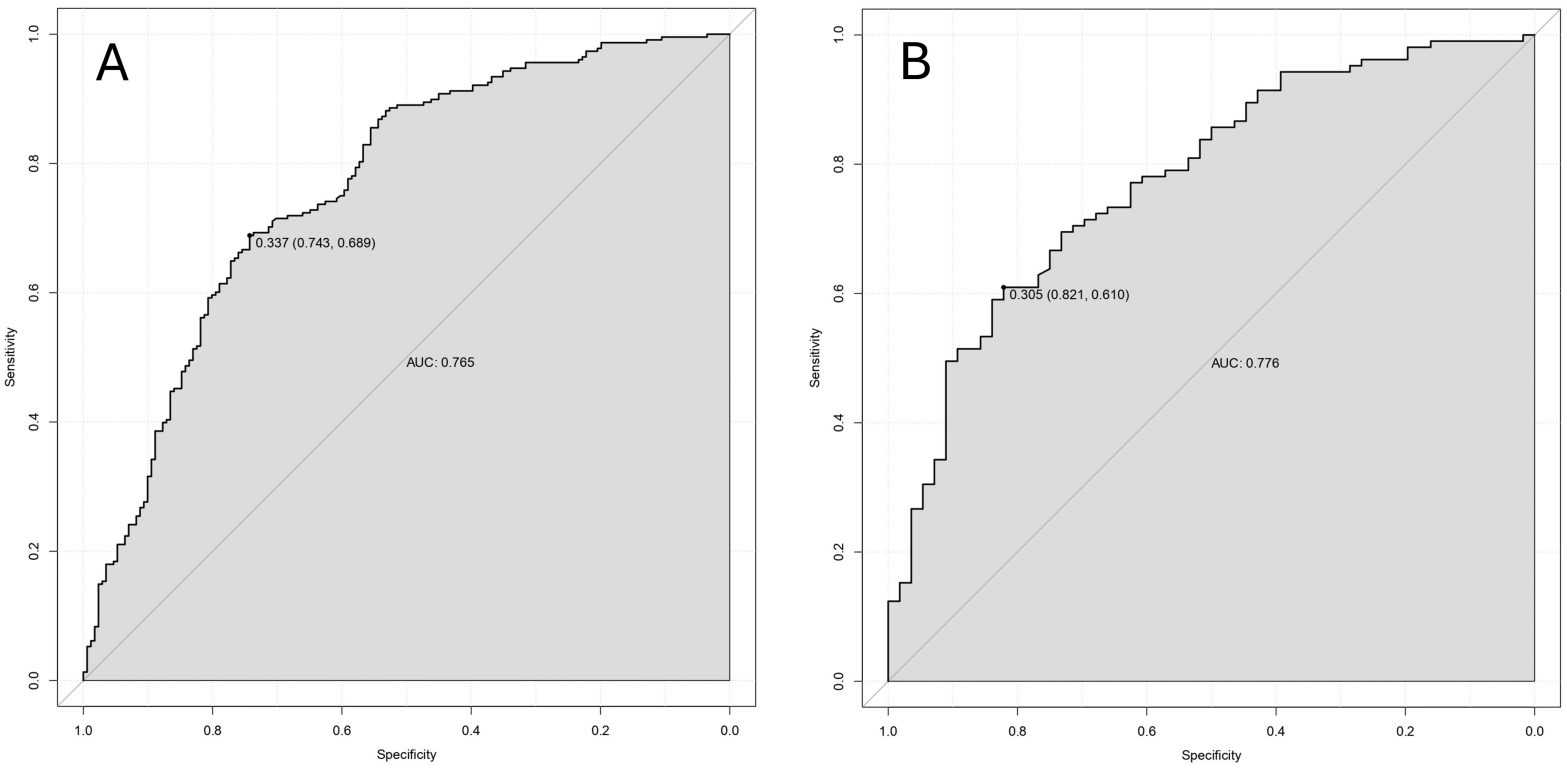
Accuracy of nomogram to predict the embryo yield for IVF/ICSI patients with endometriosis by ROC curves. ROC curves in training set **(A)** and validation set **(B)**.

**Figure 5:**
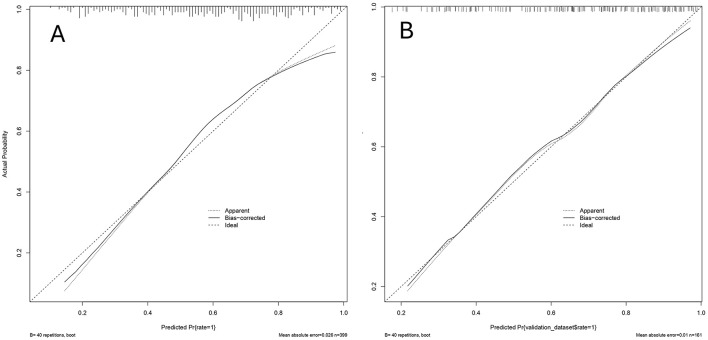
Discriminative power of nomogram to predict the embryo yield for IVF/ICSI patients with endometriosis. Calibration curves of the training set **(A)** and the validation set **(B)**.

**Figure 6:**
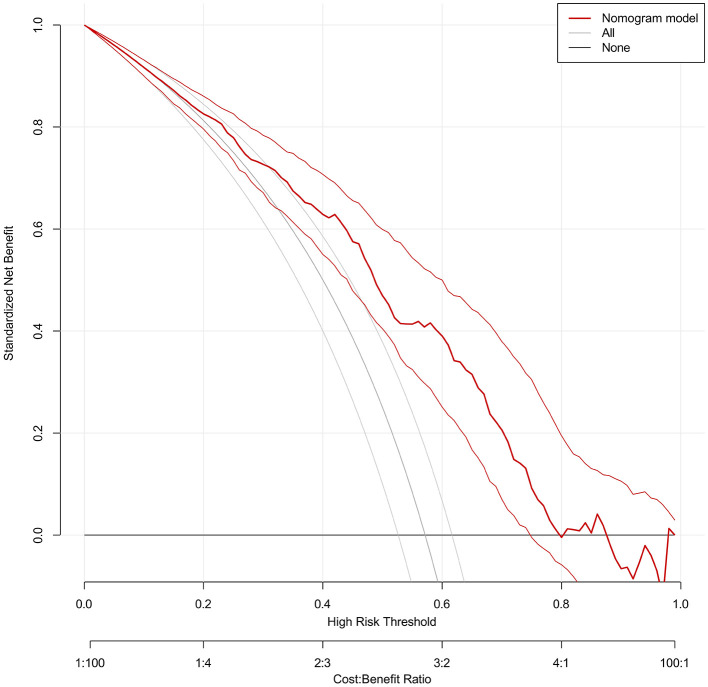
The clinical decision curves of the nomogram for embryo yield in endometriosis patients.

A predictive nomogram was constructed based on the independent predictors identified from the multivariate logistic regression analysis (Figure 3). The model exhibited strong and consistent discriminative ability, with an AUC of 0.765 (95% CI: 0.749–0.769) in the training set and 0.776 (95% CI: 0.747–0.789) in the validation set (Figure 4). Calibration curves for both cohorts showed excellent agreement between the predicted probabilities and actual observations (Figure 5). Furthermore, decision curve analysis demonstrated a superior net benefit of the nomogram across a wide range of threshold probabilities, underscoring its clinical utility (Figure 6).

### 3.3 Analysis of interaction between bilateral endometriomas and down-regulation

The binary logistic regression model developed from the training set did not identify a statistically significant additive or multiplicative interaction between the presence of bilateral endometriomas and the use of GnRH agonist down-regulation.

#### 3.3.1 Restricted cubic spline and subgroup analysis for AFC

**Figure 7:**
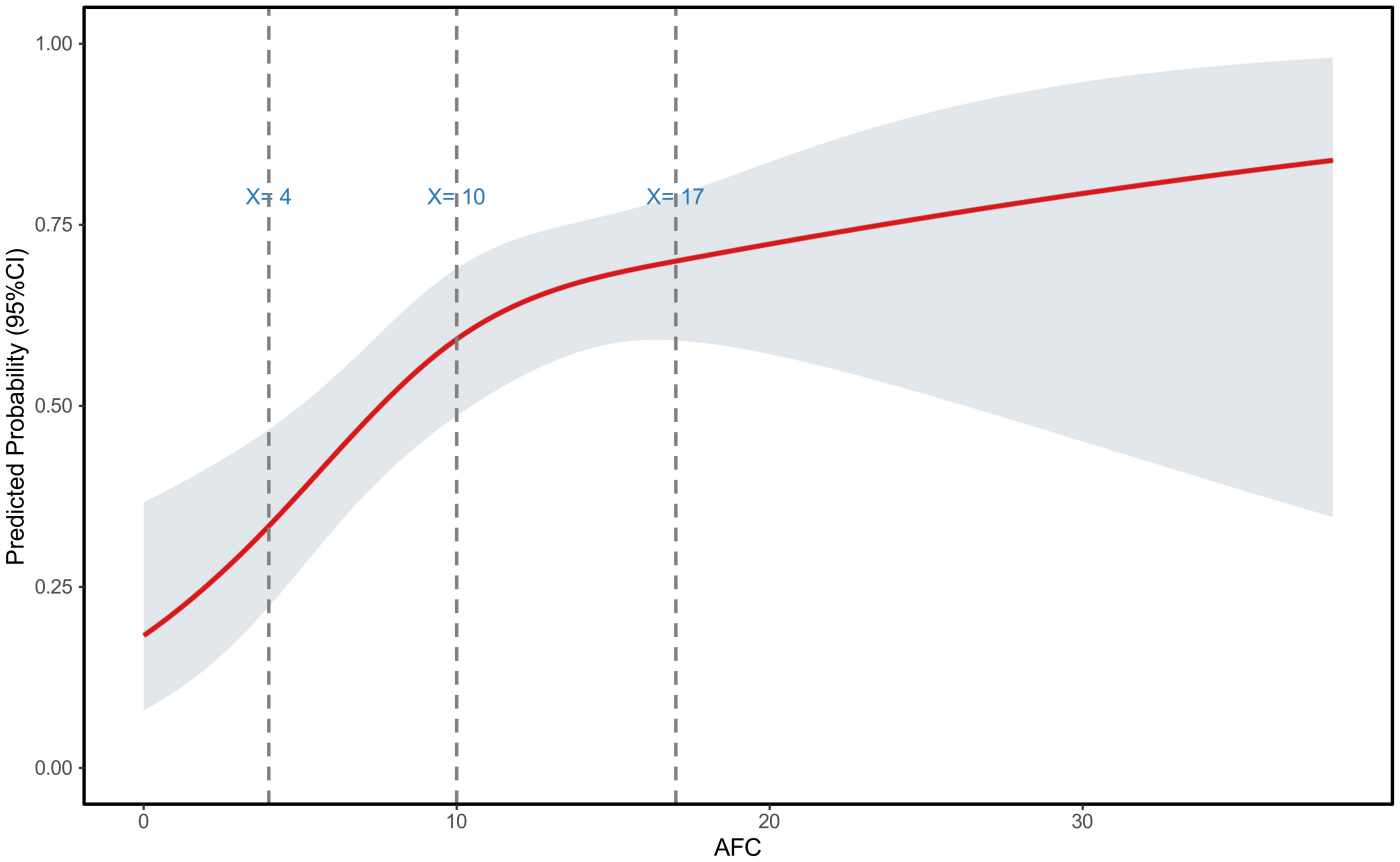
Restrictive cubic spline plot of AFC in patients with endometriosis.

A RCS was fitted with AFC as the independent variable, revealing three inflection points at AFC values of 4, 10, and 17 (Figure 7). Subsequent subgroup analysis based on these thresholds demonstrated significant differences among the three subgroups in the number of oocytes retrieved, MII oocytes, and high-quality embryos. However, no statistically significant differences were observed in the fertilization rate, 2PN rate, cleavage rate, Day 3 usable embryo rate, Day 3 high-quality embryo rate, usable blastocyst rate, or high-quality blastocyst rate.

#### 3.3.2 Restricted cubic spline and subgroup analysis for AMH

**Figure 8:**
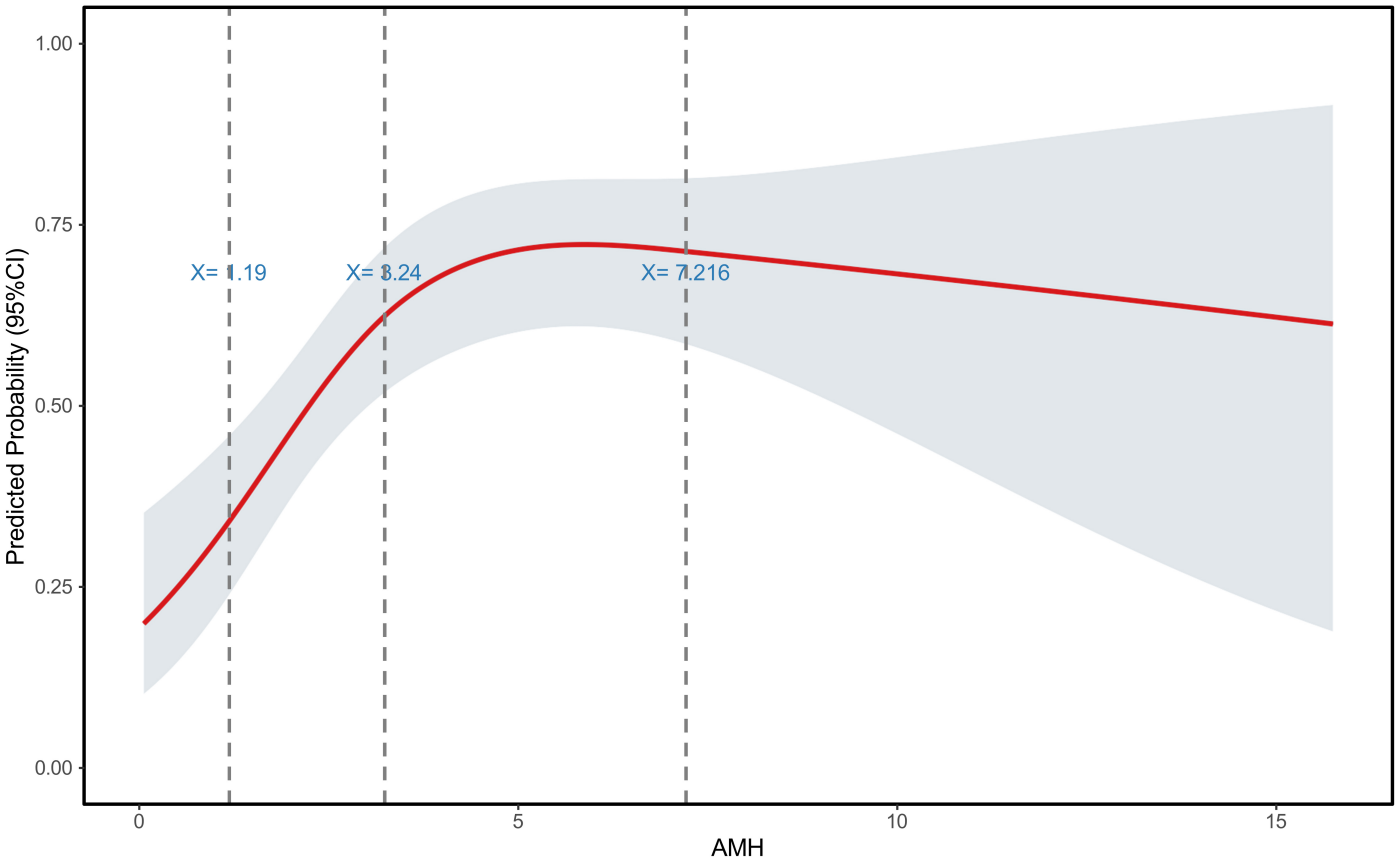
Restrictive cubic spline plot of AMH in patients with endometriosis.

An RCS analysis of AMH identified three inflection points at 1.19, 3.24, and 7.216 ng/ml (Figure 8). Subgroup analysis stratified by these thresholds revealed significant differences in oocyte yield, MII oocyte count, and the number of high-quality embryos. With the exception of the cleavage rate, which showed a significant difference, all other embryological outcomes—including fertilization rate, 2PN rate, Day 3 usable embryo rate, Day 3 high-quality embryo rate, usable blastocyst rate, and high-quality blastocyst rate—showed no statistically significant differences among the subgroups.

### 3.4 Impact of endometriosis phenotypes on embryo quality

A total of 195 patients were stratified into three groups: isolated DIE (*n* = 59), isolated OMA (*n* = 68), and combined OMA with DIE (*n* = 68). Baseline characteristics, including age, BMI, infertility type, baseline hormone levels, AFC, AMH, and the rate of coexisting male factors, were comparable across all groups, indicating well-balanced baseline profiles. The presence of ovarian endometrioma was associated with a significant reduction in the number of oocytes retrieved and MII oocytes, with the most pronounced decrease observed in the combined OMA-DIE group. Significant differences among the three groups were identified in the 2PN rate, cleavage rate, Day 3 usable embryo rate, and Day 3 high-quality embryo rate. In contrast, no significant differences were observed in the fertilization rate, usable blastocyst rate, or high-quality blastocyst rate (Table 2).

**Table 2:** Baseline characteristics and treatment outcomes of different phenotypes patients.

Characteristics	Isolated DIE (*N* = 59)	Isolated OMA (*N* = 68)	Combined OMA-DIE (*N* = 68)	*p*-value
Age (year)	32.00 (30.00, 34.00)	31.50 (30.00, 35.00)	31.50 (30.0, 35.00)	0.918
BMI (kg/m2)	21.20 (19.20, 23.60)	21.50 (19.55, 24.20)	20.70 (19.70, 23.00)	0.530
Previous pregnancy				0.566
Yes, *n* (%)	62.71 (37/59)	66.18 (45/68)	57.35 (39/68)	
No, *n* (%)	37.29 (22/59)	33.82 (23/68)	42.65 (29/68)	
Duration of infertility (year)	3.00 (2.00, 4.00)	3.00 (2.00, 4.00)	2.00 (1.00, 3.00)	0.007
bFSH (mIU/mL)	7.56 (6.67, 9.10)	7.95 (6.28, 9.92)	7.81 (6.53, 8.74)	0.682
bLH (mIU/mL)	4.69 (3.63, 5.80)	4.12 (2.93, 5.50)	4.67 (3.29, 6.05)	0.268
bE2 (pg/mL)	39.2 (29.74, 53.00)	39.60 (30.49, 60.00)	39.60 (29.61, 54.00)	0.865
AFC (*n*)	12.00 (7.00, 15.00)	10.00 (7.00, 13.00)	9.00 (5.00, 12.00)	0.052
AMH (ng/mL)	3.84 (2.16, 5.68)	3.25 (1.84, 4.48)	2.71 (1.78, 4.05)	0.071
Male factor (%)	22.03 (13/59)	20.59 (14/68)	26.47 (18/68)	0.700
Down-regulation				0.978
Yes, *n* (%)	54.24 (32/59)	55.88 (38/68)	55.88 (38/68)	
No, *n* (%)	55.76 (27/59)	44.12 (30/68)	44.12 (30/68)	
Surgery (%)				< 0.001
Yes, *n* (%)	77.97 (46/59)	44.12 (30/68)	70.59 (48/68)	
No, *n* (%)	22.03 (13/59)	55.88 (38/68)	29.41 (20/68)	
Oocytes retrieved (*n*)	14.00 (10.00, 18.00)	11.00 (6.25, 14.00)	10.00 (5.00, 14.75)	0.002
Fertilization rate (%)	73.29 (623/850)	80.77 (542/761)	72.94 (550/754)	0.617
Number of 2PN embryos (%)	65.41 (556/850)	61.89 (471/761)	59.02 (445/754)	0.030
Cleavage rate (%)	81.79 (548/670)	78.87 (462/585)	85.40 (468/548)	0.019
Metaphase II oocyte (n)	11.00 (8.00, 17.00)	8.50 (5.25, 12.00)	7.00 (4.00, 11.75)	< 0.001
Viable embryo rate on day 3 (%)	81.62 (413/506)	88.22 (352/399)	72.58 (323/445)	< 0.001
Good-quality embryos rate on day 3 (%)	60.47 (306/506)	82.27 (246/399)	53.26 (237/445)	0.024
Viable blastocyst rate (%)	76.28 (195/253)	79.12 (144/182)	82.89 (155/187)	0.327
Good blastocyst development rate (%)	53.92 (158/253)	63.19 (115/182)	63.10 (118/187)	0.789

BMI, Body Mass Index; AFC, antral follicle counts; GnRH, Gonadotropin-releasing hormone; AMH, anti-Müllerian hormone; bFSH, basal Follicle Stimulating Hormone; bLH, basal Luteinizing hormone; bE2, basal Estradiol; 2PN, two pronuclei.

### 3.5 Phenotype-specific effects under non-down-regulation protocols

Under non-down-regulation protocols (*n* = 87), baseline characteristics were balanced across the isolated DIE (*n* = 27), isolated OMA (*n* = 30), and combined OMA-DIE (*n* = 30) groups. The presence of ovarian endometrioma was associated with significantly lower AFC, oocyte yield, and MII oocyte count. Furthermore, significant intergroup differences were observed in fertilization rate, cleavage rate, and Day 3 usable embryo rate. However, no significant differences were found in the 2PN rate, Day 3 high-quality embryo rate, usable blastocyst rate, or high-quality blastocyst rate (Table 3).

**Table 3:** Baseline characteristics and treatment outcomes of patients under non-down-regulation protocols.

Characteristics	Isolated DIE (*n* = 27)	Isolated OMA (*n* = 30)	Combined OMA-DIE (*n* = 30)	*p*-value
Age (year)	33.00 (30.00, 35.00)	33.00 (30.75, 37.75)	34.00 (30.75, 40.00)	0.435
BMI (kg/m2)	20.80 (18.80, 22.20)	21.70 (20.08, 23.38)	22.05 (20.15, 24.88)	0.174
Previous pregnancy				0.726
Yes, *n* (%)	59.26 (16/27)	50.00 (15/30)	50.00 (15/30)	
No, *n* (%)	40.74 (11/27)	50.00 (15/30)	50.00 (15/30)	
Duration of infertility (year)	3.00 (2.00, 5.00)	3.50 (2.00, 5.00)	2.00 (1.00, 3.00)	0.001
bFSH (mIU/mL)	8.47 (6.70, 9.21)	8.19 (6.31, 11.12)	7.94 (6.84, 10.25)	0.974
bLH (mIU/mL)	4.98 (3.55, 5.92)	3.75 (2.62, 5.16)	4.36 (3.04, 5.67)	0.211
bE2 (pg/mL)	39.20 (29.74, 51.00)	48.33 (32.01, 74.10)	39.80 (31.75, 49.25)	0.189
AFC (*n*)	11.00 (6.00, 15.00)	9.50 (6.00, 12.25)	6.00 (4.75, 10.00)	0.019
AMH (ng/mL)	2.64 (1.47, 6.65)	2.01 (1.10, 3.92)	2.01 (1.15, 2.96)	0.076
Male factor (%)	29.63 (8/27)	26.67 (8/30)	33.33 (10/30)	0.852
Surgery (%)				0.347
Yes, *n* (%)	70.37 (19/27)	56.67 (17/30)	73.33 (22/30)	
No, *n* (%)	29.63 (8/27)	43.33 (13/30)	26.67 (8/30)	
Oocytes retrieved (*n*)	12.00 (7.00, 16.00)	7.00 (4.75, 12.25)	5.00 (3.00, 9.00)	0.001
Fertilization rate (%)	69.29 (264/381)	69.76 (173/248)	78.50 (168/214)	0.040
Number of 2PN embryos (%)	62.99 (240/381)	65.32 (162/248)	64.95 (139/214)	0.806
Cleavage rate (%)	75.59 (223/295)	77.95 (152/195)	86.22 (144/167)	0.024
Metaphase II oocyte (*n*)	9.00 (5.00, 14.00)	6.50 (4.00, 11.00)	4.00 (3.00, 8.00)	0.003
Viable embryo rate on day 3 (%)	82.21 (171/208)	89.66 (130/145)	92.04 (104/113)	0.022
Good-quality embryos rate on day 3 (%)	63.94 (133/208)	61.38 (89/145)	61.95 (70/113)	0.873
Viable blastocyst rate (%)	80.37 (86/107)	79.66 (47/59)	80.77 (42/52)	0.989
Good blastocyst development rate (%)	65.42 (70/107)	64.41 (38/59)	57.69 (30/52)	0.624

BMI, Body Mass Index; AFC, antral follicle counts; GnRH, Gonadotropin-releasing hormone; AMH, anti-Müllerian hormone; bFSH, basal Follicle Stimulating Hormone; bLH, basal Luteinizing hormone; bE2, basal Estradiol; 2PN, two pronuclei.

### 3.6 Impact of down-regulation on embryo quality across endometriosis phenotypes

#### 3.6.1 Phenotype-specific analysis under down-regulation protocol

A total of 107 patients undergoing down-regulation protocols were stratified into three phenotypic groups: isolated DIE (*n* = 31), isolated ovarian endometrioma (OMA, *n* = 38), and combined OMA-DIE (*n* = 38). Baseline characteristics—including age, BMI, infertility type, baseline hormone profiles, AFC, AMH levels, and the proportion of coexisting male factors—were comparable across groups, with the exception of a statistically significant difference in the rate of previous surgical intervention. Analysis of embryological outcomes revealed significant intergroup differences in 2PN fertilization rate, cleavage rate, MII oocyte yield, Day 3 usable embryo rate, and Day 3 high-quality embryo rate. In contrast, no statistically significant differences were observed in total oocyte retrieval, fertilization rate, usable blastocyst rate, or high-quality blastocyst rate (Table 4).

**Table 4:** Baseline characteristics and treatment outcomes of patients under down-regulation protocols.

Characteristics	Isolated DIE (*N* = 31)	Isolated OMA (*N* = 38)	Combined OMA-DIE (*N* = 38)	*p*-value
Age (year)	31.00 (29.25, 34.00)	30.00 (29.00, 34.00)	31.00 (29.00, 34.00)	0.832
BMI (kg/m2)	21.35 (19.55, 24.15)	21.35 (19.08, 24.60)	20.15 (19.40, 21.98)	0.125
Previous pregnancy				0.940
Yes, *n* (%)	64.52 (20/31)	60.53 (23/38)	63.16 (24/38)	
No, *n* (%)	35.48 (11/31)	39.47 (15/38)	36.84 (14/38)	
Duration of infertility (year)	2.00 (2.00, 3.00)	3.00 (2.00, 3.00)	2.00 (1.75, 4.00)	0.751
bFSH (mIU/mL)	7.30 (6.57, 8.85)	7.63 (6.24, 9.23)	7.17 (6.05, 8.03)	0.479
bLH (mIU/mL)	4.60 (3.70, 5.63)	4.32 (3.48, 5.65)	4.86 (3.46, 6.26)	0.528
bE2 (pg/mL)	39.00 (29.38, 53.00)	37.00 (26.42, 55.00)	39.25 (27.29, 55.50)	0.807
AFC (*n*)	13.00 (8.25, 15.00)	10.00 (7.00, 13.00)	10.50 (8.75, 14.25)	0.172
AMH (ng/mL)	4.10 (2.73, 5.51)	3.57 (2.84, 4.83)	3.55 (2.39, 4.81)	0.446
Male factor (%)	16.13 (5/31)	21.05 (8/38)	21.05 (8/38)	0.844
**Surgery (%)**				0.001
Yes, *n* (%)	87.10 (27/31)	44.74 (17/38)	68.42 (26/38)	
No, *n* (%)	12.90 (4/31)	55.26 (21/38)	31.58 (12/38)	
Oocytes retrieved (*n*)	16.00 (12.00, 19.75)	11.00 (9.00, 16.75)	12.50 (9.75, 16.25)	0.071
Fertilization rate (%)	76.10 (347/456)	71.93 (369/513)	70.74 (382/540)	0.146
Number of 2PN embryos (%)	66.67 (304/456)	60.23 (309/513)	56.67 (306/540)	0.005
Cleavage rate (%)	86.23 (313/363)	65.13 (254/390)	85.04 (324/381)	< 0.001
Metaphase II oocyte (*n*)	13.00 (10.25, 18.00)	10.00 (6.75, 14.25)	9.50 (7.00, 13.25)	0.017
Viable embryo rate on day 3 (%)	81.82 (234/286)	87.40 (222/254)	65.96 (219/332)	< 0.001
Good-quality embryos rate on day 3 (%)	59.09 (169/286)	61.81 (157/254)	50.30 (167/332)	< 0.001
Viable blastocyst rate (%)	74.66 (109/146)	78.86 (97/123)	83.70 (113/135)	0.178
Good blastocyst development rate (%)	60.27 (88/146)	62.60 (77/123)	65.19 (88/135)	0.697

BMI, Body Mass Index; AFC, antral follicle counts; GnRH, Gonadotropin-releasing hormone; AMH, anti-Müllerian hormone; bFSH, basal Follicle Stimulating Hormone; bLH, basal Luteinizing hormone; bE2, basal Estradiol; 2PN, two pronuclei.

#### 3.6.2 Effect of down-regulation in patients with isolated DIE

A matched cohort of 62 DIE patients—s31 receiving down-regulation and 31 without—demonstrated balanced baseline characteristics across all evaluated parameters. The down-regulation group exhibited a statistically significant improvement in cleavage rate (*P* < 0.05). No significant differences were observed in oocyte yield, fertilization rate, 2PN rate, MII oocyte count, Day 3 usable embryo rate, Day 3 high-quality embryo rate, usable blastocyst rate, or high-quality blastocyst rate (Table 5).

**Table 5:** Baseline characteristics and treatment outcomes of patients with die under down-regulation protocols.

Characteristics	Down-regulation group (*N* = 31)	Non-down-regulation group (*N* = 31)	*p*-value
Age (year)	31.00 (29.00, 33.00)	31.00 (29.00, 33.00)	0.744
BMI (kg/m2)	21.20 (19.20, 24.30)	20.90 (18.80, 22.70)	0.338
Previous pregnancy			0.600
Yes, *n* (%)	41.94 (13/31)	32.26 (10/31)	
No, *n* (%)	58.06 (18/31)	67.74 (21/31)	
Duration of infertility (year)	3.00 (2.00, 4.00)	3.00 (2.00, 5.00)	0.391
bFSH (mIU/mL)	7.23 (6.02, 8.29)	8.03 (6.67, 9.05)	0.057
bLH (mIU/mL)	4.83 (3.51, 5.93)	4.86 (3.21, 5.80)	0.849
bE2 (pg/mL)	43.00 (27.80, 57.50)	42.00 (30.30, 57.00)	0.965
AFC (*n*)	12.00 (7.00, 16.00)	12 (7, 15)	0.816
AMH (ng/mL)	4.15 (3.10, 6.65)	3.82 (1.80, 6.69)	0.371
Male factor (%)	9.68 (3/31)	19.35 (6/31)	0.473
Surgery (%)	80.65 (25/31)	74.19 (23/31)	0.544
Oocytes retrieved (*n*)	13.00 (10.00, 19.00)	14.00 (8.00, 19.00)	0.316
Fertilization rate (%)	73.26 (348/475)	71.97 (321/446)	0.661
Number of 2PN embryos (%)	63.58 (302/475)	63.90 (285/446)	0.919
Cleavage rate (%)	84.46 (326/386)	78.32 (271/346)	0.033
Metaphase II oocyte (*n*)	12.00 (9.00,18.00)	11.00 (7.00,17.00)	0.316
Viable embryo rate on day 3 (%)	78.50 (230/293)	81.03 (205/253)	0.464
Good-quality embryos rate on day 3 (%)	58.36 (171/293)	61.66 (156/253)	0.433
Viable blastocyst rate (%)	78.75 (126/160)	84.38 (108/128)	0.224
Good blastocyst development rate (%)	63.13 (101/160)	68.75 (88/128)	0.318

BMI, Body Mass Index; AFC, antral follicle counts; GnRH, Gonadotropin-releasing hormone; AMH, anti-Müllerian hormone; bFSH, basal Follicle Stimulating Hormone; bLH, basal Luteinizing hormone; bE2, basal Estradiol; DIE, deep infiltrating endometriosis; 2PN, two pronuclei.

#### 3.6.3 Effect of down-regulation in patients with ovarian endometrioma

Among 156 patients with ovarian endometrioma (78 per group), baseline characteristics were well-balanced between those receiving down-regulation and those managed without. The down-regulation group demonstrated statistically superior outcomes in fertilization rate, 2PN rate, and MII oocyte yield (all *P* < 0.05). However, no significant intergroup differences were observed in cleavage rate, Day 3 usable embryo rate, Day 3 high-quality embryo rate, usable blastocyst rate, or high-quality blastocyst rate (Table 6).

**Table 6:** Baseline characteristics and treatment outcomes of patients with endometrioma under down-regulation protocols.

Characteristics	Down-regulation group (*N* = 78)	Non-down-regulation group (*N* = 78)	*p*-value
Age (year)	31.50 (29.00, 34.00)	31.00 (29.00, 33.00)	0.470
BMI (kg/m2)	21.40 (19.50, 24.10)	21.40 (19.15, 22.88)	0.505
Previous pregnancy			0.861
Yes, *n* (%)	29.49 (23/78)	30.77 (24/78)	
No, *n* (%)	70.51 (55/78)	69.23 (54/78)	
Duration of infertility (year)	2.50 (2.00, 4.00)	2.25 (2.00, 4.00)	0.996
bFSH (mIU/mL)	7.51 (6.24, 9.37)	7.92 (6.43, 10.05)	0.572
bLH (mIU/mL)	4.18 (3.13, 5.18)	4.50 (3.46, 6.40)	0.158
bE2 (pg/mL)	38.90 (28.00, 57.25)	40.00 (31.70, 55.08)	0.460
AFC (*n*)	9.00 (7.00, 13.00)	9.50 (6.00, 14.00)	0.987
AMH (ng/mL)	3.37 (2.44, 4.82)	2.66 (1.97, 4.41)	0.100
Male factor (%)	39.74 (31/78)	38.46 (30/78)	0.870
Surgery (%)	62.82 (49/78)	66.67 (52/78)	0.615
Bilateral ovarian endometriomas			0.579
Yes, *n* (%)	23.08 (18/78)	26.92 (21/78)	
No, *n* (%)	76.92 (60/78)	73.08 (57/78)	
Oocytes retrieved (*n*)	11.00 (7.00, 16.25)	10.50 (5.00, 16.00)	0.182
Fertilization rate (%)	73.22 (719/982)	62.82 (556/885)	< 0.001
Number of 2PN embryos (%)	63.24 (621/982)	56.38 (499/885)	0.003
Cleavage rate (%)	81.00 (622/768)	78.31 (491/627)	0.215
Metaphase II oocyte (*n*)	10.00 (6.00, 14.00)	7.50 (5.00, 12.00)	0.043
Viable embryo rate on day 3 (%)	86.88 (470/541)	84.93 (372/438)	0.383
Good-quality embryos rate on day 3 (%)	58.04 (314/541)	59.13 (259/438)	0.730
Blastocyst formation rate (%)	68.04 (247/363)	68.14 (201/292)	0.980
Viable blastocyst rate (%)	83.00 (205/247)	84.93 (372/438)	0.652
Good blastocyst development rate (%)	60.73 (150/247)	66.67 (134/201)	0.194

BMI, Body Mass Index; AFC, antral follicle counts; GnRH, Gonadotropin-releasing hormone; AMH, anti-Müllerian hormone; bFSH, basal Follicle Stimulating Hormone; bLH, basal Luteinizing hormone; bE2, basal Estradiol; 2PN, two pronuclei.

### 3.7 Impact of surgical intervention on embryo quality in patients with ovarian endometrioma

Following propensity score matching to balance baseline characteristics, 168 patients with ovarian endometrioma were allocated into two groups: a surgical intervention group (*n* = 84) and a non-surgical management group (*n* = 84). The two groups demonstrated comparable baseline profiles, including age, BMI, duration of infertility, AFC, AMH levels, baseline hormone profiles, distribution of ovarian stimulation protocols, types of infertility, and proportion of bilateral ovarian endometrioma. Embryological assessment revealed that the non-surgical group achieved a significantly higher 2PN rate compared to the surgical group. However, no statistically significant differences were observed between the two groups in the number of oocytes retrieved, fertilization rate, cleavage rate, MII oocyte yield, Day 3 usable embryo rate, Day 3 high-quality embryo rate, usable blastocyst rate, or high-quality blastocyst rate (Table 7).

**Table 7:** Compare the impact of surgical intervention on embryo quality in patients with ovarian endometrioma.

Characteristics	Surgery and IVF (*N* = 84)	IVF (*N* = 84)	*p*-value
Age (year)	31.00 (28.25, 35.00)	31.00 (29.25, 34.00)	0.829
BMI (kg/m2)	21.50 (19.50, 23.30)	21.70 (19.42, 23.78)	0.907
Previous pregnancy			1.000
Yes, *n* (%)	39.29 (33/84)	39.29 (33/84)	
No, *n* (%)	60.71 (51/84)	60.71 (51/84)	
Duration of infertility (year)	2.75 (2.00, 4.88)	2.00 (2.00, 3.00)	0.172
bFSH (mIU/mL)	8.09 (6.55, 9.98)	7.96 (6.38, 9.86)	0.630
bLH (mIU/mL)	4.07 (3.20, 5.98)	4.13 (3.23, 5.28)	0.879
bE2 (pg/mL)	40.30 (30.00, 58.75)	39.70 (29.92, 58.00)	0.783
AFC (*n*)	8.00 (6.00, 12.00)	8.00 (6.00, 12.00)	0.996
AMH (ng/mL)	2.50 (1.45, 4.32)	2.64 (1.62, 3.78)	0.802
Male factor (%)	26.19 (22/84)	35.71 (30/84)	0.182
GnRH agonist			0.351
Yes, *n* (%)	47.62 (40/84)	40.48 (34/84)	
No, *n* (%)	52.38 (44/84)	59.52 (50/84)	
Bilateral ovarian endometriomas			0.584
Yes, *n* (%)	21.43 (18/84)	25.00 (21/84)	
No, *n* (%)	78.57 (66/84)	75.00 (63/84)	
Oocytes retrieved (*n*)	9.50 (5.00, 14.75)	8.50 (5.00, 14.00)	0.932
Fertilization rate (%)			
< 35 year	66.30 (484/730)	72.76 (553/760)	0.007
≥35 year	72.09 (124/172)	62.83 (71/113)	0.100
**Number of 2PN embryos (%)**			
< 35 year	56.44 (412/730)	63.42 (482/760)	0.011
≥35 year	60.47 (104/172)	53.98 (61/113)	0.278
Cleavage rate (%)	77.66 (504/649)	81.87 (569/695)	0.054
Metaphase II oocyte (*n*)	6.50 (4.00, 12.00)	7.00 (4.00, 11.00)	0.913
Viable embryo rate on Day 3 (%)	87.39 (388/444)	86.32 (429/497)	0.628
Good-quality embryos rate on Day 3 (%)	59.91 (266/444)	61.37 (305/497)	0.648
Blastocyst formation rate (%)	71.53 (201/281)	71.25 (233/327)	0.940

BMI, Body Mass Index; AFC, antral follicle counts; GnRH, Gonadotropin-releasing hormone; AMH, anti-Müllerian hormone; bFSH, basal Follicle Stimulating Hormone; bLH, basal Luteinizing hormone; bE2, basal Estradiol; 2PN, 2 pronuclei.

### 3.8 Impact of bilateral ovarian endometrioma on embryo quality and quantity

A total of 83 patients diagnosed with bilateral ovarian endometrioma were included in the analysis. When compared to patients with other endometriosis phenotypes, this subgroup exhibited statistically significant reductions in three key parameters: the number of oocytes retrieved, the yield of MII oocytes, and the number of high-quality embryos. Aside from a statistically significant difference in cleavage rate, no other embryological parameters—including fertilization rate, 2PN rate, Day 3 usable embryo rate, Day 3 high-quality embryo rate, usable blastocyst rate, and high-quality blastocyst rate—demonstrated significant differences (Table 8).

**Table 8:** Compare the impact of bilateral ovarian endometrioma on embryo quality and quantity.

Characteristics	Bilateral ovarian endometriomas groups (*N* = 83)	(*N* = 479)	*p*-value
Oocytes retrieved (*n*)	7.0 (4.0, 14.0)	12.0 (7.0, 18.0)	< 0.001
Fertilization rate (%)	73.22 (577/788)	70.17 (4,399/6,299)	0.077
Number of 2PN embryos (%)	61.42 (484/788)	59.90 (3,755/6,269)	0.411
Cleavage rate (%)	84.30 (494/586)	78.84 (3,830/4,858)	0.002
Metaphase II oocyte (*n*)	7.0 (4.0, 10.0)	9.0 (5.0, 14.0)	< 0.001
Viable embryo rate on day 3 (%)	86.60 (362/418)	83.40 (2,869/3,440)	0.094
Good-quality embryos rate on day 3 (%)	62.44 (261/418)	58.78 (2,022/3,400)	0.150
Viable blastocyst rate (%)	87.57 (155/177)	82.38 (1,300/1,578)	0.082
Good blastocyst development rate (%)	70.62 (125/177)	63.37 (1,000/1,578)	0.057
Good blastocyst numbers (*n*)	2.0 (1.0, 4.0)	3.0 (2.0, 5.0)	0.002

2PN, 2 pronuclei.

## 4 Discussion

To date, no well-validated model exists for predicting pregnancy outcomes in patients with endometriosis. Although several diagnostic models have been developed to estimate pregnancy rates in this population and have demonstrated certain predictive capability, their generalizability is limited due to the heterogeneous impact of different endometriosis phenotypes on reproductive outcomes. Given the considerable compensatory capacity of the ovary, most patients with endometriosis—except those with severe bilateral ovarian endometrioma—can still achieve a clinically relevant number of embryos. This observation suggests that cumulative pregnancy rate and live birth rate may represent more meaningful endpoints than pregnancy rate per cycle. According to a study by Paula et al., patients with stage I/II endometriosis achieved a cumulative pregnancy rate of 64.5% and a live birth rate of 51.6% after four IVF/ICSI cycles, compared to 44.8% and 32.8%, respectively, for those with stage III/IV disease. Moreover, after three embryo transfers, the live birth rates approached approximately 50% for stage I/II and 30% for stage III/IV patients (21). According to the latest European Society of Human Reproduction and Embryology guidelines, the traditional fixed-number criterion for defining recurrent implantation failure has been superseded by an approach based on the cumulative predicted probability of pregnancy (22). The guidelines recommend individualized assessment using predictive models, with a recommended threshold of 60%. However, to date, no such model has been developed for this purpose. For patients with a low cumulative pregnancy rate, further evaluation is recommended. Given that patients with endometriosis often exhibit a reduced oocyte yield, the outcome variable was defined as the attainment of three high-quality embryos. Based on these findings, we develop a predictive model for the likelihood of obtaining a sufficient number of high-quality embryos—a surrogate endpoint for estimating cumulative pregnancy and live birth rates. Using a stepwise regression approach, four variables were identified as independent predictors: AFC, AMH level, the presence of bilateral ovarian endometrioma, and the use of GnRH agonist pretreatment. The final model demonstrated satisfactory discriminative ability and calibration.

Endometriosis impairs fertility through multiple interconnected mechanisms, including the establishment of a pelvic inflammatory milieu, diminished ovarian reserve, compromised endometrial receptivity, and potential genetic influences. Previous meta-analyses investigating IVF outcomes in endometriosis patients reported that those with ovarian endometrioma exhibit reduced oocyte yield, decreased numbers of MII oocytes, and fewer embryos obtained, while maintaining comparable pregnancy and live birth rates to other infertility etiologies (12). A recent meta-analysis further substantiated that patients with ovarian endometrioma yield significantly fewer oocytes (WMD −2.25, 95% CI −3.43 to −1.06) and MII oocytes (WMD −4.64, 95% CI −5.65 to −3.63) compared to healthy controls (23). Consequently, ovarian reserve impairment directly affects the success rate of each ART stimulation cycle (24, 25). Dalila Invernici et al. (26) proposed that confounding factors such as age and ovarian reserve may account for negative findings in some studies. After meticulous matching for age, stimulation protocol, and AMH levels, their analysis revealed no significant difference in the number of high-quality embryos between women with and without endometriosis. However, patients with endometriosis demonstrated a higher incidence of poor ovarian response and reduced oocyte retrieval. These findings collectively validate the detrimental impact of endometriosis on folliculogenesis. While the rASRM classification has been widely employed to assess endometriosis severity, its clinical utility is limited by the frequent coexistence of different phenotypic manifestations—peritoneal, deep infiltrating, and ovarian—making it challenging to quantify their individual contributions. Nevertheless, a Cochrane review comparing surgical and ultrasound diagnosis reported high accuracy for transvaginal ultrasonography in detecting ovarian endometrioma (sensitivity 0.93, specificity 0.96) and deep infiltrating endometriosis (sensitivity 0.79, specificity 0.94) (27). This diagnostic reliability, coupled with advancements in IVF technology, has expanded clinical management options. Our results demonstrate that the presence of ovarian endometrioma significantly reduces oocyte yield and MII oocyte numbers compared to isolated DIE, with a parallel decline in ovarian reserve parameters. This effect is further exacerbated when ovarian endometrioma coexists with DIE, a pattern consistently observed regardless of GnRH agonist pretreatment. Although statistically significant differences in oocyte and embryo quality parameters were detected among the three phenotypic groups, their clinical relevance remains uncertain and warrants verification through larger-scale studies. The current evidence primarily supports the conclusion that endometriosis predominantly affects oocyte and embryo quantity rather than quality. This interpretation aligns with the meta-analysis by Dongye et al. (28), which found comparable rates of high-quality embryos (RR = 1.00; 95% CI, 0.94–1.06), cleavage (RR = 1.00; 95% CI: 0.97–1.02), and embryo formation (RR = 1.10; 95% CI: 0.97–1.24) between endometriosis patients and controls, with no significant differences even in stage III–IV disease. These consistent findings reinforce our conclusion that the fundamental impact of endometriosis on fertility lies in quantitative rather than qualitative embryological parameters.

The impact of bilateral ovarian endometriomas on embryo quality remains a subject of ongoing debate. Reinblatt et al. compared embryo quality between 13 patients with bilateral endometriomas and 39 without, concluding that bilateral endometriomas did not significantly affect embryo quantity (29). However, this study was limited by a small sample size and exhibited a non-significant trend toward reduced embryo quality, suggesting possible type II statistical error due to inadequate power. To address this limitation, Laura et al. conducted a larger analysis comparing 39 patients with unoperated bilateral endometriomas to 78 controls, arriving at a different conclusion—bilateral endometriomas were associated with reduced embryo quantity (30). Our multivariate logistic regression results align with the latter finding, identifying bilateral endometriomas as an independent risk factor for decreased numbers of high-quality embryos. Specifically, patients with bilateral endometriomas demonstrated significantly reduced embryo yields, though no significant differences were observed in other embryological quality parameters except for cleavage rate. Further compounding the clinical challenge, Somigliana et al. reported that patients with bilateral endometriomas who underwent cystectomy prior to IVF exhibited higher cycle cancellation rates due to poor ovarian response. Despite receiving higher gonadotropin doses, these patients yielded fewer oocytes and achieved lower pregnancy and live birth rates compared to non-surgical controls (31). Collectively, these findings position bilateral ovarian endometriomas as a distinct clinical entity. The condition likely represents a more severe disease phenotype, characterized by the loss of compensatory function from a contralateral healthy ovary, further diminished ovarian reserve, and elevated surgical risks. These features create substantial challenges in clinical decision-making. Additionally, the potential benefits of GnRH agonist pretreatment warrant focused investigation in this specific subgroup. Unfortunately, the limited sample size of our cohort precluded a dedicated analysis of this clinically important population.

The optimal ovarian stimulation protocol for patients with endometriosis remains a subject of ongoing investigation without established consensus. The systematic review by Sallam et al. (17) included only three studies, two of which reported no statistically significant differences in outcomes. Subsequently, Georgiou et al. (19) conducted a more comprehensive systematic review incorporating eight randomized controlled trials. However, due to the frequent absence of blinding in these studies and imprecision in primary outcome measures, the authors concluded that existing evidence was insufficient to support the routine use of GnRH agonists for improving IVF outcomes in endometriosis patients. Cao et al. (18) reported in their meta-analysis that ultra-long GnRH agonist protocols significantly improved clinical pregnancy rates in patients with stage III–IV endometriosis (RR = 2.04, 95% CI: 1.37–3.04) within RCTs, while no significant benefit was observed for those with stage I–II disease. Notably, this effect was not replicated in non-randomized studies, leading the authors to refrain from making definitive clinical recommendations. While numerous studies have suggested potential benefits of GnRH agonist pretreatment, theoretical concerns persist regarding the risk of poor ovarian response, particularly in patients with ovarian endometriomas. This has prompted consideration of GnRH antagonist protocols as a viable alternative. In the present study, after controlling for confounding factors including age, BMI, and ovarian reserve parameters, we observed that GnRH agonist down-regulation significantly improved fertilization rates, 2PN rates, and MII oocyte yields in patients with ovarian endometriomas, as well as enhanced cleavage rates in those with DIE. Furthermore, interaction analysis revealed no significant interaction terms, indicating that the effects of GnRH agonist therapy and bilateral endometriomas on embryo yield operate independently. Despite these findings suggesting potential benefits of GnRH agonist pretreatment, the advantages of antagonist protocols should not be overlooked. In large-sample scenarios, the risks associated with GnRH agonists might be obscured. Therefore, for patients with diminished ovarian reserve, treatment decisions should remain individualized, carefully weighing the potential benefits of GnRH agonist down-regulation against the flexibility and possibly lower risk of poor response offered by antagonist regimens.

Recent studies have established that surgical management of ovarian endometrioma may adversely affect ovarian reserve, even when performed by experienced surgeons (13, 32). A meta-analysis of 14 prospective studies demonstrated a significant postoperative decline in AMH levels (mean decrease: 1.77 ng/ml; 95% CI: 0.77–2.77; *P* < 0.001), with the reduction being independent of patient age (33). Concurrently, a comprehensive meta-analysis by Muzii et al. (5) encompassing 17 studies indicated that patients with unoperated ovarian endometrioma already exhibit significantly lower AMH levels compared to healthy controls or those with other benign ovarian cysts. Given these risks, the question of whether cystectomy should be routinely performed prior to IVF remains contentious. Our findings indicate that surgical intervention did not improve embryo quantity or quality in patients with ovarian endometrioma, regardless of age, after meticulous matching for both chronological age and ovarian reserve parameters. Restricted cubic spline analysis revealed a non-linear relationship between ovarian reserve markers and embryo yield, underscoring the prognostic value of these biomarkers in assessing fertility potential in endometriosis patients. The decline in IVF outcomes becomes apparent when ovarian reserve begins to be compromised (AMH < 3.24 ng/ml, AFC < 10), and this trend intensifies with further diminishment of reserve (AMH < 1.19 ng/ml, AFC < 4). To ensure adequate oocyte retrieval in endometriosis patients, comprehensive preoperative assessment and careful patient selection for surgical intervention are paramount. Current evidence-based guidelines do not recommend routine laparoscopic surgery for endometriosis-associated infertility patients without severe dysmenorrhea (16). Furthermore, vitrification technology holds strategic importance for fertility preservation in this population. Considering the dual threats of surgical iatrogenic damage and disease progression, oocyte cryopreservation represents a viable option for endometriosis patients who lack a partner, are career-focused, or have no immediate fertility plans, particularly those anticipated to undergo surgical intervention for ovarian endometrioma.

This study has several limitations that should be considered when interpreting the results. First, the absence of histological confirmation of endometriosis in a subset of patients may introduce selection bias. Second, embryo morphological grading does not account for chromosomal aneuploidy; even high-quality embryos may fail to implant or result in miscarriage due to genetic abnormalities. Future studies incorporating preimplantation genetic testing could help elucidate the relationship between endometriosis and embryonic aneuploidy. Third, the analysis did not include certain male factors that could influence embryonic development, such as semen quality and sperm DNA fragmentation rates. Additionally, the retrospective, single-center design of this study may limit the generalizability of our findings. Notwithstanding these limitations, our findings support several clinically relevant conclusions. GnRH agonist pretreatment appears to improve key IVF laboratory parameters in patients with endometriosis. Oocyte cryopreservation represents a viable fertility preservation strategy for individuals with ovarian endometrioma, particularly those with immediate fertility constraints. Surgical intervention should be considered judiciously, with special caution exercised for patients presenting with bilateral ovarian endometriomas, given the potential for further compromise of ovarian reserve.

## 5 Conclusion

In conclusion, this study identified serum AMH level, AFC, and the presence of bilateral ovarian endometriomas as independent factors influencing the yield of high-quality embryos. A predictive nomogram incorporating these factors demonstrated robust performance in both training and validation sets. Subgroup analyses revealed that ovarian endometrioma, particularly in its bilateral form or when co-existing with DIE, significantly impairs oocyte and embryo quantity. Notably, surgical intervention for endometrioma did not improve embryological outcomes compared to conservative management. These findings support the use of the developed nomogram for the early prediction of ovarian response and suggest that GnRH agonist pretreatment can be beneficial, while surgical decisions should be individualized, with oocyte cryopreservation considered a viable fertility preservation option for specific patient profiles.

## Data Availability

The raw data supporting the conclusions of this article will be made available by the authors, without undue reservation.
