# Supplementary figures and images for: Analysis of risk factors compromising embryo quality and quantity in patients with endometriosis
Source: Front Med (Lausanne). 2026 Apr 23;13:1808405. doi: 10.3389/fmed.2026.1808405 (PMC13149374; doi:10.3389/fmed.2026.1808405)

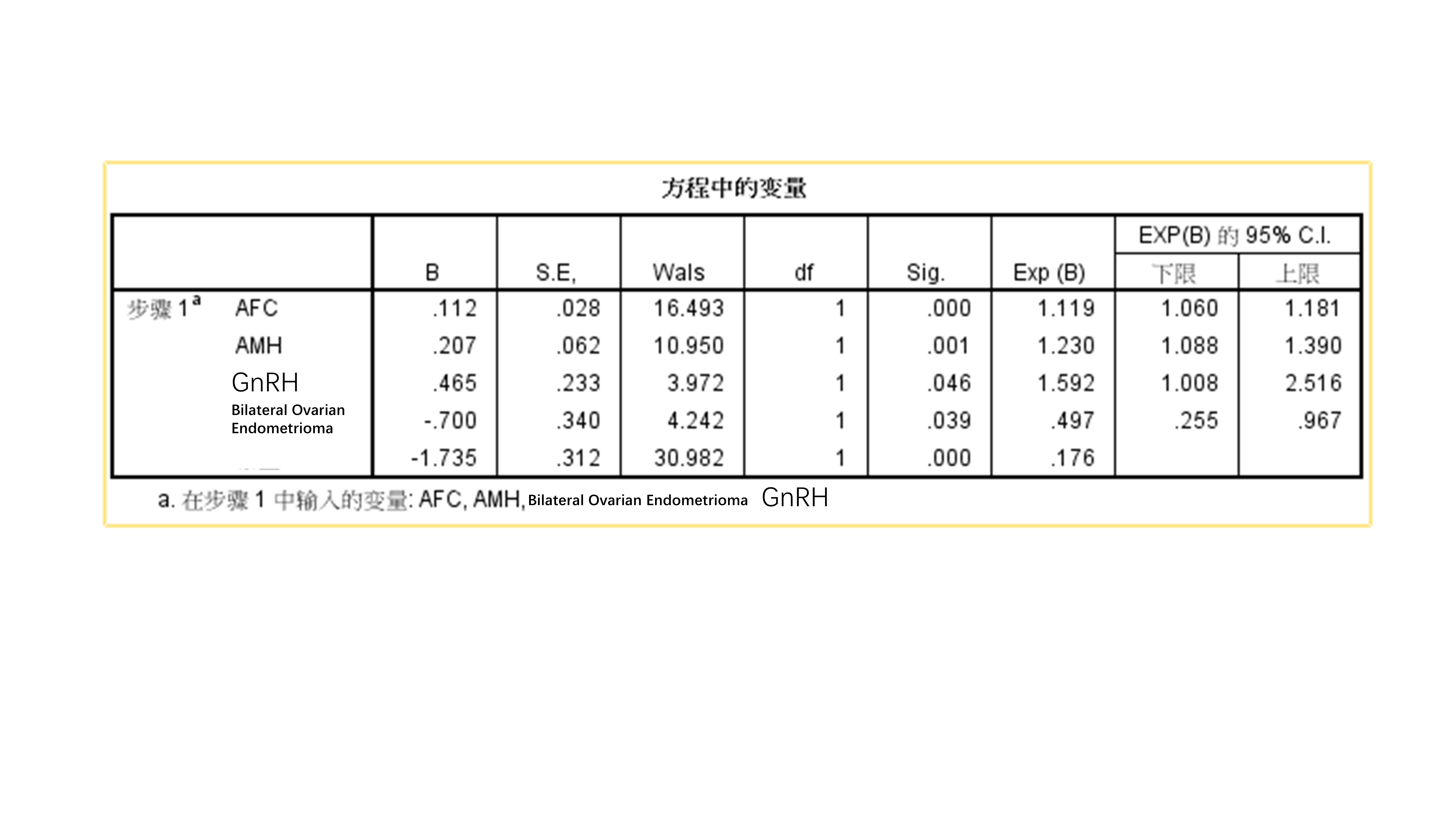

Supplement: Supplementary file 1 [file Image_1.tif]
